# Genomic analysis and selective small molecule inhibition identifies BCL-X_L_ as a critical survival factor in a subset of colorectal cancer

**DOI:** 10.1186/s12943-015-0397-y

**Published:** 2015-07-02

**Authors:** Haichao Zhang, John Xue, Paul Hessler, Stephen K. Tahir, Jun Chen, Sha Jin, Andrew J. Souers, Joel D. Leverson, Lloyd T. Lam

**Affiliations:** Oncology Research, AbbVie, 1 N Waukegan Road, North Chicago, IL 60064-6101 USA; Tumor Genomics AP10-214, Department R4CD, 1 North Waukegan Road, North Chicago, IL 60064-6098 USA

**Keywords:** BCL-X_L_, MCL-1, NOXA, BCL-X_L_ inhibitor, Colorectal cancer

## Abstract

**Background:**

Defects in programmed cell death, or apoptosis, are a hallmark of cancer. The anti-apoptotic B-cell lymphoma 2 (BCL-2) family proteins, including BCL-2, BCL-X_L_, and MCL-1 have been characterized as key survival factors in multiple cancer types. Because cancer types with *BCL2* and *MCL1* amplification are more prone to inhibition of their respectively encoded proteins, we hypothesized that cancers with a significant frequency of *BCL2L1* amplification would have greater dependency on BCL-X_L_ for survival.

**Methods:**

To identify tumor subtypes that have significant frequency of *BCL2L1* amplification, we performed data mining using The Cancer Genome Atlas (TCGA) database. We then assessed the dependency on BCL-X_L_ in a panel of cell lines using a selective and potent BCL-X_L_ inhibitor, A-1155463, and *BCL2L1* siRNA. Mechanistic studies on the role of BCL-X_L_ were further undertaken via a variety of genetic manipulations.

**Results:**

We identified colorectal cancer as having the highest frequency of *BCL2L1* amplification across all tumor types examined. Colorectal cancer cell lines with *BCL2L1* copy number >3 were more sensitive to A-1155463. Consistently, cell lines with high expression of BCL-X_L_ and NOXA, a pro-apoptotic protein that antagonizes MCL-1 activity were sensitive to A-1155463. Silencing the expression of BCL-X_L_ via siRNA killed the cell lines that were sensitive to A-1155463 while having little effect on lines that were resistant. Furthermore, silencing the expression of MCL-1 in resistant cell lines conferred sensitivity to A-1155463, whereas silencing NOXA abrogated sensitivity.

**Conclusions:**

This work demonstrates the utility of characterizing frequent genomic alterations to identify cancer survival genes. In addition, these studies demonstrate the utility of the highly potent and selective compound A-1155463 for investigating the role of BCL-X_L_ in mediating the survival of specific tumor types, and indicate that BCL-X_L_ inhibition could be an effective treatment for colorectal tumors with high BCL-X_L_ and NOXA expression.

## Background

Cancer is a genetic disease that arises from the accumulation of somatic gene alterations. One way to identify key genes in cancer is to examine genomic regions that undergo frequent alterations. Recent technological advances in characterizing these alterations make it possible to identify genes that are essential for the initiation and/or survival of cancer, and thus encode potential therapeutic targets.

Evasion of apoptosis is a hallmark of cancer cells. One mechanism of apoptotic pathway deregulation is via upregulation of the anti-apoptotic BCL-2 family members. Apoptotic pathway proteins belong to a family of BCL-2 Homology (BH)-domain-containing proteins comprising three classes: 1) multi-domain anti-apoptotic (BCL-2, BCL-X_L_, BCL-W, BFL-1/A1, and MCL-1), 2) multi-domain pro-apoptotic (BAX, BAK), and 3) BH3-only pro-apoptotic (BID, BIM, BAD, BIK, NOXA, PUMA, BMF, and HRK). The BH3-only proteins contain a single BH3 domain and are bound by specific anti-apoptotic proteins [1]. For example, BCL-2 and BCL-X_L_ bind and antagonize BIM but not NOXA. In contrast, MCL-1 and A1 bind and antagonize NOXA but not BAD protein. Other BH3 domain proteins such as BIM and PUMA are bound and antagonized by all anti-apoptotic proteins. BAX and BAK are known as the “effectors”. Once activated, these proteins oligomerize on the outer mitochondrial membrane and induce pore formation; this results in the release of cytochrome c and other pro-apoptotic proteins that ultimately carry out the cell death mechanism.

The role of anti-apoptotic BCL-2 family proteins in various cancers has been well studied [2]. BCL-2 was initially identified from the breakpoint of the t(14;18) chromosomal translocation found in over 60 % of indolent B cell non-Hodgkin’s lymphoma [3–6]. In addition to the vast majority of follicular lymphomas, many germinal center B cell (GCB) subtype diffuse large B cell lymphomas (DLBCL) also exhibit the t(14;18) chromosomal translocation [7–9]. *BCL2* amplification is also detected in many hematologic malignancies such as the activated B cell-like (ABC) subtype of DLBCL [10]. Not surprisingly, cell lines with the translocation or amplification are more sensitive to the selective BCL-2 inhibitor ABT-199 [11].

*MCL1* was reported to be amplified in 10.9 % of tumor samples analyzed, spanning multiple cancer subtypes [12]. Fluorescence in situ hybridization (FISH) of the *MCL1* region identified lung and breast cancers as having significantly higher frequencies of focal amplification, suggesting that these tumors depend on MCL-1 for survival. This is supported by multiple studies demonstrating that cell lines with *MCL1* amplification are sensitive to siRNA knockdown of *MCL1* [12, 13].

BCL-X_L_ has been implicated as a key survival factor in numerous solid tumors [2]. Based on the evidence that cancer types with *BCL2* and *MCL1* amplification are more prone to inhibition of their encoded proteins, we hypothesized that cancers with a significant frequency of *BCL2L1* amplification are more dependent on BCL-X_L_ for survival. In this study, we identified colorectal cancer as having a significant incidence of *BCL2L1* amplification. We then dissected the role of BCL-X_L_ in colorectal cancer cell lines using a selective small-molecule inhibitor of BCL-X_L_ and a variety of genetic manipulations.

## Materials and methods

### Reagents

BCL-X_L_ inhibitor A-1155463 and navitoclax were synthesized at AbbVie, Inc. (North Chicago, IL). All the siRNAs were purchased from Dharmacon (Lafayette, CO).

### Cell culture, transfection, and cell-based assays

Colorectal cell lines (ATCC) were cultured in RPMI (Invitrogen, Carlsbad, CA) supplemented with 10 % fetal bovine serum (FBS) (Invitrogen), 1 % sodium pyruvate (Invitrogen), and 4.5 g/L glucose (Sigma, MO), or DMEM (Invitrogen) supplemented with 10 % FBS. All the lines were maintained in a humidified chamber at 37 °C containing 5 % CO_2_.

LS1034, SW1417, GEO, and RKO cells were transfected in 6-well plates with siRNAs using Lipofectamine 2000 according to the manufacturer’s instructions (Invitrogen). A final concentration of 20 nM siRNA was used in all cases. The sense sequences of the BCL-X^L^ siRNA used is ACAAGGAGAUGCAGGUAUUUU (Dharmacon). The sense sequences of the MCL-1 siRNAs used is GCATCGAACCATTAGCAGATT (Dharmacon). The cells were then grown in medium without antibiotic before harvesting for western blotting analysis. LS1034 cells were transfected at 1.5–2.5 × 10^4^ cells/100 μl in 96-well tissue culture plates with 20 nM Noxa siRNA pool (Dharmacon). The cells were grown in medium without antibiotic before harvesting.

Cells were treated with increasing concentration of A-1155463. Cells were assayed for viability after 72 h using the CellTiter-Glo luminescent cell viability assay according to the manufacturer’s protocol (Promega, Madison, WI). Results were normalized to cells without treatment. EC50 was calculated using the GraphPad Prism software (La Jolla, CA).

### Western blot analysis

Cell lysates were prepared in RIPA buffer (Sigma) plus protease inhibitor cocktail (Roche). 20 μg of total protein was resolved on a 12 % SDS polyacrylamide gel and probed with anti-BCL-X_L_ (Epitomics, Burlingame, CA), anti-MCL-1 (Epitomics), anti-BCL-2 (BD), anti-BIM (Epitomics), anti-actin and anti-NOXA (Abcam, Cambridge, MA). Antibody against tubulin (Santa Cruz Biotechnology, Inc., Santa Cruz, CA) was used as a loading control.

### Fluorescence-activated cell sorting (FACS) analysis

LS1034 cells were treated with DMSO or 200 nM A-1155463, with or without 50 μM Z-VAD caspase inhibitor (Santa Cruz Biotechnology, Inc.) for 72 h. DNA content was measured by flow cytometry to determine the effect of the inhibitors on the cell cycle and cell death. Following treatment, cells were spun down, the medium was removed and the cells were resuspended in staining solution (50 μg propidium iodide, 40 U/ml RNase, 0.1 % triton X-100 in PBS) at a cell concentration of 1 × 10^6^ cells/ml. The cells were stored in the dark at room temperature for at least 30 min or up to 24 h at 4 °C before the DNA content was determined by flow cytometry using a FACS Calibur (BD Biosciences, San Jose, CA).

## Results

**Fig. 1:**
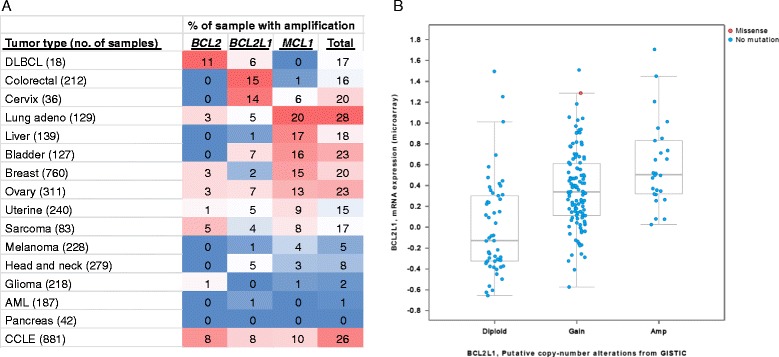
Cross-cancer tumor sample analysis identified colorectal cancer as having the highest frequency of *BCL2L1* amplification. **a** Cross-cancer alteration summary for *BCL2*, *BCL2L1* and *MCL1* based on data obtained from cBioPortal for Cancer Genomics. **b** Correlation between gene expression and copy number alteration of *BCL2L1* in 212 colorectal cancer samples

BCL-X_L_ has been implicated as a key survival factor in numerous solid tumors. Since cancer types with *BCL2* and *MCL1* amplification are more prone to inhibition of these proteins, we reasoned that significant frequency of *BCL2L1* amplification in a particular cancer type may indicate dependency on BCL-X_L_ for survival. To identify cancer types with a significant amplification of *BCL2L1*, we utilized The cBio Cancer Genomics Portal (http://cbioportal.org), an open-access resource for interactive exploration of multidimensional cancer genomics data sets from more than 5,000 tumor samples from 20 cancer studies [14]. We surveyed the percentage of copy number alternations of *BCL2*, *BCL2L1* and *MCL1* among a majority of these tumor samples (Fig. 1a) and found that colorectal cancer has the highest percentage of *BCL2L1* gain/amplification among all cancer types. Out of 212 samples, 15 % have *BCL2L1* amplification, while *MCL1* amplification is found in only 1 %. *BCL2* amplifications were not observed, although homozygous deletion is observed in 1 % of colorectal cancer samples (data not shown). Furthermore, there is a good correlation between *BCL2L1* copy number gain/amplification and gene expression as determined by RNAseq and microarray analysis (Fig. 1b and data not shown). Cervical cancer also has a high percentage of *BCL2L1* gains/amplifications (Fig. 1a) although the sample numbers are limited. Consistent with other studies, we found that DLBCL has the highest percentage of *BCL2* amplification (11 %) and lung adenocarcinoma has the highest percentage of *MCL1* amplification (20 %) (Fig. 1a). These data indicated that each tumor type has a selective dependency on a particular anti-apoptotic protein for survival and colorectal and cervical tumors are potentially more dependent on BCL-X_L_ for survival.

**Fig. 2:**
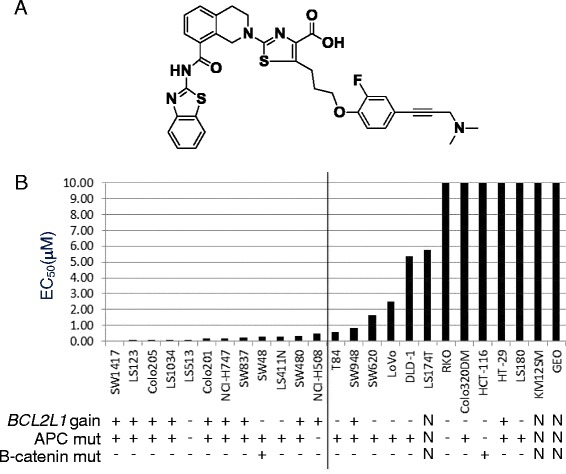
Colon cancer cell lines with *BCL2L1* gain are more sensitive to BCL-X_L_ inhibitor. **a** Structure of the potent and specific BCL-X_L_ inhibitor A-1155463. **b** Colorectal cell lines were treated with increasing concentrations of A-1155463. Cells were assayed for viability after 72 h. Results were normalized to cells without treatment. Each cell line was treated with A-1155463 in at least 3 different experiments and the average EC_50_ is presented. *BCL2L1* copy number gain >3 is indicated with + or -. Mutation status of APC and β-catenin is also indicated with + or -. These data are taken from the CCLE database [24]. N denotes cases where data were not available

To test this hypothesis, we selected a panel of 25 colorectal cancer cell lines with known copy number of *BCL2L1*_._ We first investigated the sensitivity of these colorectal cell lines to treatment with the selective BCL-X_L_ inhibitor, A-1155463 ([15] and Fig. 2a). This recently reported small molecule is more potent in inhibiting BCL-X_L_ than navitoclax but shows negligible activity against BCL-2 or MCL-1, thus making it an excellent tool for dissecting BCL-X_L_ cancer biology. A-1155463 demonstrated strong growth inhibition of over half of the colorectal cell lines (Fig. 2b) as defined by EC_50_ values ≤0.5 μM in the presence of 10 % FBS. Consistent with its lower potency against BCL-X_L_, navitoclax induced cell death in the same cell lines, although at higher concentrations (data not shown).

Previously, we and others have shown that copy number (CN) gain, protein and mRNA expression of BCL-2 family members is a crucial determinant of sensitivity to BH3 mimetics such as ABT-737, an inhibitor of BCL-2, BCL-X_L_, and BCL-W, and ABT-199, a specific inhibitor of BCL-2 [11, 16–18]. Herein we report for the first time that cell lines with *BCL2L1* gains are sensitive to selective BCL-X_L_ inhibition. Among the 11 lines with *BCL2L1* gain (CN > 3), 9 are sensitive to A-1155463 (EC_50_ < 0.5 μM). In contrast, only 3 out of 11 lines with no CN gain of *BCL2L1* are sensitive to A-1155463 (Fig. 2b).

**Fig. 3:**
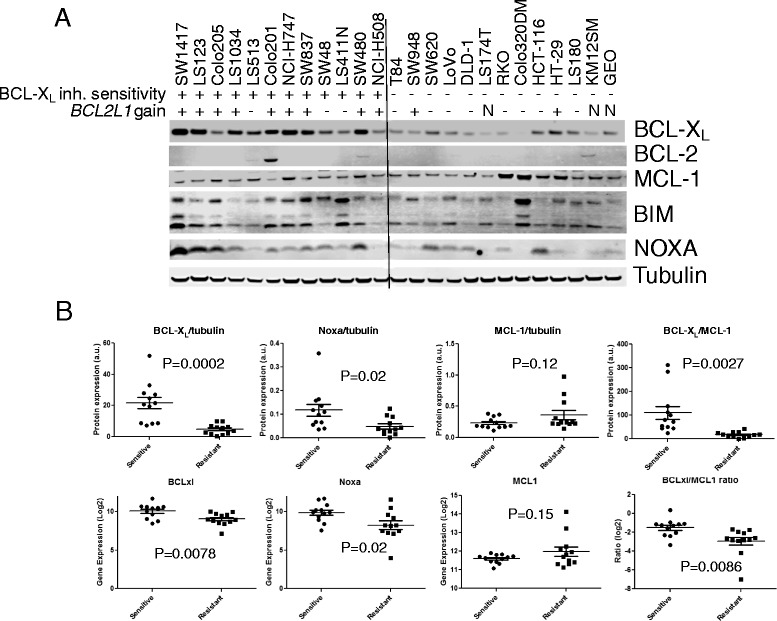
Colon cancer cell lines with high BCL-X_L_ and NOXA are more sensitive to BCL-X_L_ inhibitor. **a** Protein expression of BCL-2 family members in colorectal cancer cell lines. *BCL2L1* copy number gains >3 is indicated with + or -. **b** Protein and mRNA expression of BCL-X_L_, NOXA, MCL-1, and ratio of BCL-X_L_ /MCL-1 in A-1155463-sensitive vs. resistant colorectal cancer cell lines

To determine if expression of BCL-2 family members plays a role in the sensitivity of colorectal cell lines to A-1155463, we collected lysates from these cell lines and performed western blotting analysis. Colorectal cell lines within our panel expressed varying levels of MCL-1 and BCL-X_L_ (Fig. 3a). In general, there was an inverse relationship between the expression of MCL-1 versus NOXA and BCL-X_L_. We observed a strong correlation (0.68) between cell lines with *BCL2L1* gain and BCL-X_L_ protein expression. In addition, sensitive lines had higher protein expression of BCL-X_L_ (*p* = 0.0002) and NOXA (*p* = 0.02), higher BCL-X_L_ to MCL-1 ratio (*p* = 0.0027), and a trend towards lower expression of MCL-1, although not statistical significant (*p* = 0.12). The Pearson correlation between log EC_50_ and expression of BCL-X_L_, NOXA, MCL-1, and BCL-X_L_/MCL-1 was 0.75, 0.653, 0.363, and 0.637, respectively. The Spearman correlation between EC_50_ and expression of BCL-X_L_, NOXA, MCL-1, and BCL-X_L_/MCL-1 was 0.789, 0.749, 0.277, and 0.795, respectively. BCL-2 expression was detected in a single line, consistent with other studies showing that BCL-2 expression is less common in solid tumor cell lines [19]. To confirm these findings, we performed correlation analyses with mRNA expression data. There was a strong correlation between protein and mRNA expression for MCL-1 and BCL-X_L._ Additionally, sensitive lines had higher expression of *BCL2L1* (*p* = 0.0078) and NOXA (*p* = 0.02), a higher *BCL2L1 to MCL1* ratio (*p* = 0.0086), and a trend towards lower expression of *MCL1* mRNA, although not statistical significant (*p* = 0.15) (Fig. 3b). The correlation coefficients for *BCL2L1* and *MCL1* versus sensitivity were −0.41 and 0.56, respectively, while the correlation coefficient for the *BCL2L1* to *MCL1* ratio was −0.56. These data indicate that *BCL2L1* and NOXA expression may play important roles in determining sensitivity to selective BCL-X_L_ inhibitors.

Two recent manuscripts reported that BCL-X_L_ is regulated by the β-catenin pathway. In an unbiased screen of 242 genomically characterized cancer cell lines with an Informer Set of 354 small molecules, cell lines with β-catenin mutation were reported to be amongst the most sensitive to navitoclax (ABT-263), an inhibitor of BCL-2, BCL-X_L_ and BCL-W [20]. In a separate study, β-catenin-active cancers were reported to be dependent on a signaling pathway involving the transcriptional regulator YAP1 and the transcription factor TBX5, both of which form a complex with β-catenin [21]. This complex was further shown to be important for regulating BCL-X_L_ and the anti-apoptotic gene *BIRC5*. APC is part of a destruction complex that mediates the ubiquitin-targeted proteolysis of β-catenin. Mutations in APC destabilize this complex and lead to the accumulation of β-catenin, which can then translocate to the nucleus and aberrantly activate the transcription of key drivers of oncogenesis [22]. Interestingly, a small subgroup of colorectal tumors with wild-type APC has point mutations in β-catenin that allow it to escape degradation [23]. Since BCL-X_L_ was described to be regulated by the β-catenin pathway and multiple components of the pathway are highly mutated in colorectal cancer, we determined whether a correlation exists between these mutations and the activity of inhibitor A-1155463 in the colorectal cancer cell lines. While the majority of these lines have mutations in APC, only two lines have β-catenin mutations according to the Cancer Cell Line Encyclopedia (CCLE) [24]. Contrary to recent reports, we found no correlation between APC or β-catenin mutation status and dependence on BCL-X_L_ for survival as revealed by sensitivity to A-1155463 (Fig. 2b) or navitoclax (data not shown). This could be explained by differences in cell lines between the studies. In particular, our studies focus on colon cell lines only. It is also possible that the disparity results from the lack of appropriately selective small molecules in the study by Basu et al. [20].

**Fig. 4:**
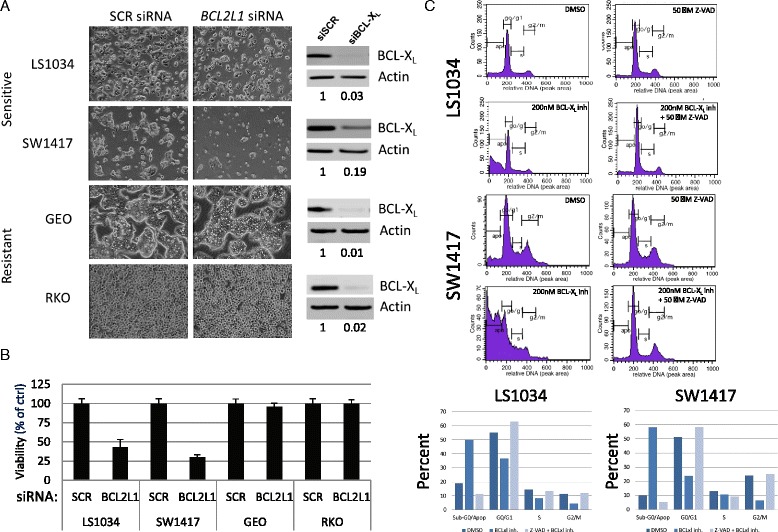
A-1155463 sensitivity correlates with the effect of BCL-X_L_ knockdown and apoptosis. *BCL2L1* or control siRNA was transfected into two A-1155463-sensitive cell lines (LS1034 and SW1417) and two resistant cell lines (GEO and RKO). **a** Viability was determined after 72 h by microscopy. Western blotting showing the degree of BCL-X_L_ knock-down by *BCL2L1* siRNAs. Levels of BCL-X_L_ knockdown were measured by densitometry and shown as BCL-X_L_/actin. **b** Viability was determined after 72 h with CellTiter Glo reagent. **c** BCL-X_L_ inhibitor induces apoptosis through activation of caspase-3/7 pathway. LS1034 and SW1417 cells were treated with DMSO or 200 nM A-1155463, with or without 50 μM Z-VAD caspase inhibitor for 24 h. DNA content was measured by flow cytometry to determine the effect of the inhibitors on the cell cycle and cell death. The percentage of cells in each phase of the cell cycle was plotted

To confirm the survival dependence on BCL-X_L_ within the colorectal cell line panel, we silenced its expression via small interfering RNA (siRNA) oligonucleotides specific to *BCL2L1* in two sensitive (LS1034 and SW1417) and two resistant (RKO and GEO) colorectal cell lines. The two sensitive lines also exhibit *BCL2L1* gain. *BCL2L1* siRNA effectively knocked down BCL-X_L_ protein expression completely in three cell lines, and ~80 % in SW1417 cells (Fig. 4a). However, the viability of only the A-1155463-sensitive colorectal cell lines was greatly reduced after transfecting *BCL2L1* siRNA (defined as >50 % reduction). In contrast, there was minimal effect on the viability of the inhibitor-resistant cell lines, thus confirming that BCL-X_L_ is critical for survival of a subgroup of colorectal cell lines. The viability is also confirmed using another assay that quantifies the ATP present in culture as an indicator of metabolically active cells (Fig. 4b). We found that the viability measured with this method is similar to that as shown in Fig. 4a, confirming that these A-1155463-sensitive colorectal cell lines indeed depend on BCL-X_L_ for survival.

To assess whether the BCL-X_L_ inhibitor induced cell death in sensitive colorectal cell lines via apoptosis, we evaluated the cell cycle distribution of LS1034 and SW1417 cells after treatment with A-1155463 in the presence or absence of the caspase-3/7 inhibitor Z-VAD. As shown in Fig. 4c, A-1155463 treatment led to substantial accumulation of sub-G0/apoptosis cells (>50 %). This effect was completely abrogated in the presence of Z-VAD, indicating that cell killing occurs via the activation of caspase-3/7 pathway.

**Fig. 5:**
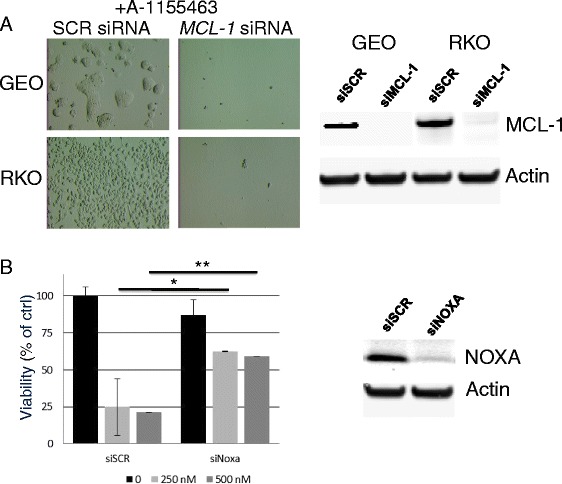
Silencing *MCL1* sensitizes colorectal cells to BCL-X_L_ inhibitor while silencing NOXA renders resistance to BCL-X_L_ inhibitor. **a** *MCL1* or control siRNA were transfected into RKO and GEO cells in the presence of A-1155463. Viability was determined after 48 h by microscopy. Western blotting showing the degree of MCL-1 knockdown by *MCL1* siRNAs in GEO and RKO cells. **b** NOXA or control siRNA was transfected into LS1034 cells in the presence or absence of A-1155463. Viability was determined after 48 h. Results were normalized to cells transfected with scrambled siRNA control and presented as mean +/− SD (n = 3). *p < 0.01, **p < 0.005. Western blotting showing the degree of NOXA knockdown by NOXA siRNA pool

We investigated the role of MCL-1 in determining sensitivity to the BCL-X_L_ inhibitor in two resistant colorectal cell lines (RKO and GEO) by silencing the expression of MCL-1 with siRNA. While silencing MCL-1 alone had no effect (data not shown), MCL-1 knockdown in the presence of A-1155463 afforded complete cell killing (Fig. 5a). Similar effects were observed with an additional resistant line, HT-29 (data not shown). These data indicate that MCL-1 plays a role in modulating sensitivity to BCL-X_L_ inhibitors in colorectal cancer cell lines.

NOXA is a pro-apoptotic protein that antagonizes MCL-1 activity. Because NOXA expression is higher in the sensitive lines (Fig. 3a), we sought to assess its role in determining sensitivity to BCL-X_L_ inhibitors. We thus asked whether silencing NOXA would rescue sensitive colorectal lines from treatment with A-1155463. Transfection of LS1034 cells with NOXA siRNA rendered the cells insensitive to A-1155463 treatment (Fig. 5b), indicating that NOXA function, likely involving its inhibition of MCL-1, is required to sensitize this cell line to BCL-X_L_ inhibition. Similar results were observed in another BCL-X_L_ inhibitor-sensitive line, SW1417 (data not shown).

## Discussion

In this study, we set out to first identify cancer types that show high levels of *BCL2L1* amplification using genomic mining. Our second major aim was to investigate the dependency of corresponding cell lines on BCL-X_L_ for survival. To this end, we used not only siRNA but a recently disclosed small molecule inhibitor of BCL-X_L_ that shows high potency and selectivity against other pro-survival proteins. Our study identified colorectal and cervical cancers as having the most frequent *BCL2L1* amplification_._ Further studies showed that the majority of colorectal cancer cell lines with a gain in *BCL2L1* are sensitive to the selective BCL-X_L_ inhibitor A-1155463 as well as to siRNA-medited knockdown of *BCL2L1*. Although the gains are typically modest (3–4.4 copies), it should be noted that CN gain of other anti-apoptotic genes such as *MCL1* is also modest in other tumors that nevertheless become dependent on these genes for survival [12]. In addition, the sensitive lines exhibit higher protein and mRNA expression of BCL-X_L_ and NOXA, and a trend towards lower protein and mRNA expression of MCL-1 than the resistant lines.

Despite a recent report that navitoclax-sensitive cell lines are enriched with β-catenin mutations [20], we found no correlation between BCL-X_L_ dependence and the presence of β-catenin or APC mutations. Possible reasons for this discrepancy could be differences in the colorectal cell lines used in the two studies, or alternatively, the fact that the highly selective BCL-X_L_ inhibitor was not available at the time of the earlier study by Basu et al. and the study was thus confounded by the mixed selectivity of the compound used [20]. Our data is consistent with those of Rosenbluh et al., who reported that β-catenin-active cancers regulate BCL-X_L_ as a survival factor [21]. In this regard, we found that sensitive lines are enriched with an active β-catenin pathway as determined by a β-catenin transcriptional reporter assay. Although this is a small sample size, preliminary data show that five out of six of the sensitive lines display active β-catenin pathway versus three out of six in the resistant group (data not shown). Collectively, our data indicate that BCL-X_L_ may be regulated through multiple pathways in colorectal cancer, including CN gain and active β-catenin signaling. The role of other pathways cannot be ruled out.

Similar to navitoclax, we found that MCL-1 is a resistance factor for the BCL-X_L_-selective inhibitor A-1155463. Mechanistically, A-1155463 and navitoclax displace pro-apoptotic protein BIM from BCL-X_L_, which leads to the activation of apoptosis [2, 25]. In resistant cells, it is likely that the BIM released by BCL-X_L_ inhibitor is readily sequestered by MCL-1, thereby inhibiting the activation of the apoptotic pathway. Indeed, it has been shown that MCL-1 and BCL-X_L_ play compensatory roles in regulating apoptosis in most solid tumor cell lines [13, 16, 26]. Our experiments further demonstrate that silencing MCL-1 can sensitize solid tumor cell lines to selective BCL-X_L_ inhibition, expanding on earlier studies with the BCL-2/BCL-X_L_ inhibitors ABT-737 and ABT-263 [27–30]. By targeting MCL-1 via mTOR inhibition, colorectal cancers with *Kras or BRAF* mutations are sensitized to BCL-2/BCL-X_L_ inhibition [31]. Our observation that silencing NOXA could rescue sensitive cells from A-1155463 also supports the role of MCL-1 as a resistance factor. NOXA is known to interact with MCL-1 and neutralize its activity by targeting this protein for degradation [1]. Thus, silencing NOXA decreases the antagonism of MCL-1, leaving it free to sequester pro-apoptotic proteins and prevent the activation of apoptosis [13].

Other gene amplifications have served as therapeutic targets for other cancers. For example, the MET and ALK tyrosine kinase inhibitor crizotinib (PF-02341066) shows differential antitumor effects in non-small cell lung and gastric cancer according to *MET* and *ALK* alterations [32]. By utilizing genomic data to identify tumors with *BCL2L1* gain, we identified colorectal cancers as being potentially more dependent on BCL-X_L_ and more susceptible to BCL-X_L_ inhibition. Indeed, by using a potent and selective BCL-X_L_ inhibitor, A-1155643, we have shown that BCL-X_L_ inhibition is sufficient to kill colon cancer cell lines expressing high levels of BCL-X_L_ and the MCL-1-neutralizing protein NOXA. Of particular interest, sensitive lines are enriched in a gain of *BCL2L1*, suggesting *BCL2L1* gain could be used as a patient stratification biomarker. This finding demonstrates the power of identifying potential new targets by examining genomic regions that undergo frequent alterations as well as the utility of highly potent and selective tool molecules. Finally, BCL-X_L_ selective inhibitors may have utility as therapeutics for the treatment of colorectal cancer, a disease for which there is significant unmet medical need.

## Conclusions

BCL-X_L_ inhibition could be an effective treatment for colorectal tumors with high BCL-X_L_ and NOXA expression. Additionally, *BCL2L1* gain may represent stratification biomarker for colorectal cancer patients undergoing treatment with a BCL-X_L_ inhibitor. Future studies should investigate the utility of *BCL2L1* gain as a stratification biomarker.
